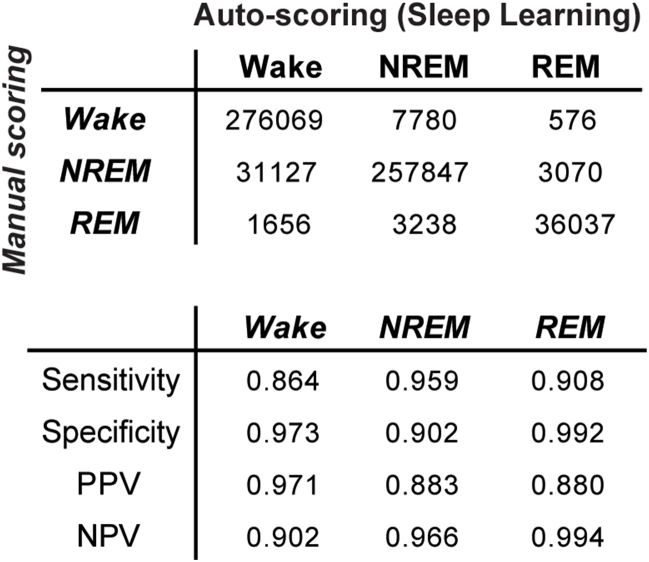# Parafacial GABAergic neurone ablation induces behavioural resistance to volatile anaesthetic-induced hypnosis without reducing sleep

**DOI:** 10.1016/j.bja.2025.02.035

**Published:** 2025-04-15

**Authors:** Toshihiro Imamura, Andrzej Z. Wasilczuk, Sarah L. Reitz, Jie Lian, Miyoko Imamura, Brendan T. Keenan, Naoki Shimizu, Allan I. Pack, Max B. Kelz

**Affiliations:** 1 Chronobiology and Sleep Institute, University of Pennsylvania Perelman School of Medicine, Philadelphia, PA, USA; 2 Division of Pulmonary and Sleep Medicine, Children's Hospital of Philadelphia, Philadelphia, PA, USA; 3 Division of Sleep Medicine, Department of Medicine, University of Pennsylvania Perelman School of Medicine, Philadelphia, PA, USA; 4 Neuroscience of Unconsciousness and Reanimation Research Alliance, University of Pennsylvania Perelman School of Medicine, Philadelphia, PA, USA; 5 Department of Anesthesiology and Critical Care, University of Pennsylvania Perelman School of Medicine, Philadelphia, PA, USA; 6 Department of Pediatrics, St. Marianna University School of Medicine, Kawasaki, Japan

**Keywords:** anaesthetic resistance, hypnosis, isoflurane, parafacial zone, sevoflurane, sleep homeostasis

## Abstract

**Background:**

It is hypothesised that general anaesthetics co-opt the neural circuits regulating endogenous sleep and wakefulness to produce hypnosis. To further probe this association, we focused on the GABAergic neurones of the parafacial zone (PZ^GABA^), a brainstem site capable of promoting non-rapid eye movement sleep.

**Methods:**

To determine whether PZ neurones are activated by a hypnotic dose of anaesthetics, c-Fos immunohistochemistry was performed. The behavioural and physiological contributions of PZ^GABA^ neurones to anaesthetic sensitivity were assessed in mice transfected with an adeno-associated virus (AAV)-driving expression of an mCherry fluorescent control or a caspase that irreversibly eliminates PZ^GABA^ neurones. EEG-defined sleep was measured in PZ^GABA^-ablated and mCherry control mice, as was the homeostatic drive to sleep after sleep deprivation.

**Results:**

Consistent with anaesthetic-induced depolarisation, hypnotic doses of isoflurane significantly increased c-Fos expression three-fold in PZ^GABA^ neurones compared with oxygen-exposed mice. PZ^GABA^-ablated mice developed significant and durable behavioural resistance to both isoflurane- and sevoflurane-induced hypnosis, with roughly 50% higher likelihood of intact righting than controls. PZ^GABA^-ablated mice emerged from isoflurane significantly faster than mCherry controls with purposeful movements. The degree of anaesthetic resistance was inversely correlated with the number of surviving PZ^GABA^ neurones. Despite confirming that PZ^GABA^ ablation reduced the potency of two distinct volatile anaesthetics behaviourally, ablation did not alter the amount of endogenous sleep or wakefulness, nor did it affect the homeostatic sleep drive after sleep deprivation, and it did not produce EEG signatures of anaesthetic resistance during isoflurane exposure.

**Conclusions:**

There was an unexpected dissociation in which destruction of up to 70–80% of PZ^GABA^ neurones was sufficient to alter anaesthetic susceptibility behaviourally without causing insomnia or altering sleep pressure. These findings suggest that PZ^GABA^ neurones are more critical to drug-induced hypnosis than to the regulation of natural sleep and arousal.

**Editor's key points**

• Neural circuits regulating sleep are engaged by anaesthetics to produce a state of unconsciousness, but key differences between sleep and anaesthesia exist.
• The role of GABAergic neurones in the parafacial zone of the brainstem, which is capable of promoting non-rapid eye movement sleep, in the effects of volatile anaesthetics on hypnosis was studied in mouse models.
• Elimination of 70–80% of parafacial zone neurones altered anaesthetic susceptibility without causing insomnia or altering sleep pressure.
• Thus, parafacial neurones are more critical to anaesthetic-induced hypnosis than to the regulation of natural sleep and arousal, which supports differences in the neurophysiological basis of physiological and anaesthetic-induced hypnosis.

General anaesthesia is essential to modern medicine, yet the precise mechanisms through which anaesthetics induce unconsciousness remain unknown. Previous evidence supports the notion that the neural circuits regulating non-rapid eye movement (NREM) sleep are engaged by anaesthetics to produce a state of unconsciousness.^1^, ^2^, ^3^, ^4^, ^5^, ^6^, ^7^ However, despite the overlap in neural circuitry between sleep and anaesthetic-induced hypnosis, key differences exist. General anaesthesia and sleep are distinct states of unconsciousness^8^, ^9^, ^10^ with contrasting physiological benefits on processes such as memory consolidation^11^ and immune function enhancement.^12^ Further, emerging research suggests nuanced differences in the homeostatic regulation of sleep and anaesthetic-induced unconsciousness. Prolonged exposure to inhaled general anaesthetics, such as isoflurane and sevoflurane, satisfies the homeostatic drive for NREM sleep.^13^^,^^14^ However, anaesthetics cannot fully substitute for sleep, as evidenced by rapid eye movement (REM) sleep rebound.^14^

Despite these differences, many studies focus on congruency between hypnotic mechanisms accompanying NREM sleep and anaesthetic-induced unconsciousness. For example, the ventrolateral preoptic (VLPO) nucleus, known for promoting sleep,^15^, ^16^, ^17^, ^18^, ^19^ is similarly implicated in anaesthesia.^3^^,^^20^, ^21^, ^22^ This circuit convergence has been demonstrated elsewhere in the hypothalamus^6^^,^^23^^,^^24^ and other brain regions, including the basal forebrain,^25^ thalamic reticular nucleus,^26^ lateral habenula,^27^ dorsal raphe,^28^ ventral tegmental area,^29^ and locus coeruleus.^30^ How these nodes regulating sleep coordinate widespread adaptations in neural activity to produce both natural and drug-induced unconscious states remains unclear. Moreover, the degree to which modulating activity of sleep-promoting neurones influences anaesthetic sensitivity and *vice versa* remains uncertain.

Here, we focus on the parafacial zone (PZ) of the brainstem, which has been suggested as a key site regulating NREM sleep. Previous studies have highlighted the role of the PZ in initiating and maintaining NREM sleep, particularly through its GABAergic neurones.^31^ Activation of GABAergic PZ neurones (PZ^GABA^) can promote consolidated periods of NREM sleep, whereas inhibition reduces NREM sleep duration and enhances wakefulness in rodents.^32^ Reciprocal projections between PZ^GABA^ neurones and wake-promoting regions, including the bed nucleus of the stria terminalis, lateral hypothalamus, ventral tegmental area, substantia nigra, pedunculopontine tegmental nucleus, dorsal raphe, locus coeruleus, and parabrachial nucleus, are considered mechanistically important for regulating arousal.^31^^,^^33^ Moreover, the wake-promoting pontine parabrachial nucleus, which receives inhibitory input from PZ^GABA^ neurones, is capable of modulating anaesthetic states as well.^34^^,^^35^

Lesions of the glutamatergic parabrachial nucleus and adjacent precoeruleus cause behavioural unresponsiveness.^36^, ^37^, ^38^ Moreover, photo-adduction of a light-reactive anaesthetic analogue within the parabrachial–precoeruleus junction markedly prolongs the duration of anaesthesia more than 20-fold.^39^ Activation of glutamatergic parabrachial neurones accelerates emergence from sevoflurane anaesthesia.^40^ Activation of parabrachial astrocytes increases wakefulness and curtails the hypnotic efficacy of isoflurane,^35^ whereas electrical stimulation of the parabrachial nucleus accelerates emergence from isoflurane anaesthesia.^41^ Neural activity within the parabrachial nucleus is suppressed during propofol and isoflurane anaesthesia and recovers during emergence.^34^ It is conceivable that the effects of experimentally modulating activity in parabrachial and other PZ^GABA^ efferent projections arise indirectly because of presumed anaesthetic-induced disinhibition of PZ^GABA^ neurones.

Given the suggested role of PZ^GABA^ neurones in modulating arousal and its established inhibitory connectivity to other wake-promoting nodes, we hypothesised that PZ^GABA^ neurones are activated by inhalation anaesthetics. We also posited that the elimination of PZ^GABA^ neurones would bias the brain towards the waking state, manifesting as both durable anaesthetic resistance and persistent insomnia. Although the former occurred, we discovered an unexpected dissociation. Ablation of up to 80% of PZ^GABA^ neurones reduced volatile anaesthetic potency behaviourally but failed to reduce NREM sleep in mice, thus highlighting a novel divergence in the circuitry regulating sleep and anaesthesia.

## Methods

Studies were approved by the Institutional Animal Care and Use Committee at the University of Pennsylvania and were conducted in accordance with NIH guidelines. Vgat-IRES-Cre;Ai6 mice were generated by breeding homozygous Vgat-IRES-Cre mice (Jackson Laboratories, Bar Harbor, ME, USA. Strain 016962) to homozygous Ai6 reporter mice (Jackson Laboratories, Strain 007906), which express a ZsGreen fluorescent reporter in Cre^+^ neurones. Righting reflex, adhesive tape removal testing, sleep–wake assessments, and assessments of isoflurane effects on cortical EEG were conducted in Vgat-IRES-Cre;Ai6 mice, aged 8–14 weeks. For adhesive tape removal testing and isoflurane EEG testing only, a subset of Vgat-IRES-Cre mice (*n*=8 of 12) were also included in the Cre-dependent mCherry control group only after confirming indistinguishable baseline isoflurane righting reflex sensitivity of Vgat-IRES-Cre;Ai6 and Vgat-IRES-Cre (lacking the Ai6 reporter gene) before AAV transfection (Supplemental Fig. S1). All mice were acclimatised to a reverse 12-h:12-h light/dark cycle (ZT0 at 19:00) for a minimum of 2 weeks before experimentation and had *ad libitum* access to food and water.

### c-Fos immunohistochemistry

Vgat-IRES-Cre;Ai6 mice were habituated to experimental chambers in 100% oxygen for 2 h a day for 3 days. On the experimental day, mice were randomly assigned to a 2-h exposure to either 100% oxygen or isoflurane 1.2 vol% mixed in 100% oxygen before sacrifice. During the exposure, mice were kept in temperature-controlled gas-tight chambers (200 ml in volume) with 200 ml min^−1^ fresh gas flow fluxing across each chamber.^42^ Mice were left undisturbed in the prone position for the duration of the exposure. After standard transcardial perfusion and tissue harvest, 40 μm coronal brain sections were processed for c-Fos expression as previously described.^43^ An Alexa Fluor 594 goat anti-rabbit secondary antibody (A11012, 1:200, Thermofisher Scientific, Waltham, MA, USA) labelled c-Fos (rabbit anti-c-Fos, 2250S, 1:1000, Cell Signalling Technologies, Danvers, MA, USA) in red, and native ZsGreen expression labelled Cre^+^ GABAergic neurones green. Sections were imaged on a confocal microscope with a 20× objective lens (SP5II, Leica Microsystems, Wetzlar, Germany). The PZ was defined as the region extending up to 500 μm dorsal to the facial nerve and spanning 700 μm laterally along the facial nerve.^31^ A experimenter blinded to treatment manually scored cells as Vgat positive, c-Fos positive, or double positive using the Cell Counter plugin within FIJI (an open source image processing package based on National Institutes of Health's ImageJ, Bethesda, MD, USA).^43^^,^^44^ Additional C57BL6/J mice (Jackon Laboratories, Strain 000664) were exposed for 2 h to 100% oxygen, 1,2-dichlorohexafluorocyclobutane (F6) 3.2 vol%, or isoflurane 1.2 vol% in the same 200 ml chambers. As a non-immobiliser, F6 does not induce hypnosis, although its structure predicts that it should.^45^ These mice were processed for c-Fos immunoreactivity identically as described above.

### Parafacial zone transfection using adeno-associated viruses

All AAV transfections targeting the parafacial zone used 30 nl of a given AAV bilaterally targeting published coordinates^46^: AP –5.35 mm, ML ±1.35 mm, and DV –5.25 mm in anaesthetised mice. To assess viral spread, AAV5-EF1a Nuc-flox(mCherry)-EGFP (Addgene, 112677, 1.2×10^13^ GC ml^−1^ Watertown, MA, USA) was injected into Vgat-IRES-Cre;Ai6 mice using established protocols.^47^ This virus drives nuclear mCherry expression in cells lacking Cre or nuclear GFP Cre-expressing cells. Four weeks after microinjection, mice were perfused and post-fixed. Brains were sectioned coronally at 40 μm and imaged using a BZ-X800 microscope (Keyence, Itasca, IL, USA). Stereotactic injections were performed using a microinjection syringe pump (Stoelting, Wood Dale, IL, USA) with a 33 G, 10 *μ*l syringe (Hamilton 80008, Hamilton Company, Reno, NV, USA).

### Creation of PZ^GABA^ ablation and mCherry control mice

Anaesthetised mice were injected bilaterally with 30 nl of AAV5-flex-taCasp3-TEVp (Addgene, 45580, 1.5×10^13^ GC ml^−1^) or AAV8-hSyn-DIO-mCherry virus (Addgene, 50459, 2.2×10^13^ GC ml^−1^) using the aforementioned stereotaxic coordinates. These groups are hereafter called PZ^GABA^-ablated (*n*=44) and mCherry controls (*n*=35).

### Righting reflex assessment of anaesthetic sensitivity

Transgenic mice underwent steady-state righting reflex to establish baseline individual anaesthetic sensitivity as previously described.^42^^,^^48^, ^49^, ^50^ Mice were exposed for 4 h to either isoflurane or sevoflurane, mixed in 100% oxygen, at the sex-specific^51^ approximate population EC_50_: isoflurane 0.60 vol% (males), isoflurane 0.75 vol% (females), sevoflurane 1.0 vol% (males), or sevoflurane 1.2 vol% (females). The righting reflex was assessed every 3 min during the last 2 h of the exposure.^43^^,^^50^^,^^51^ Mice were then randomly assigned to either the PZ^GABA^-ablated or mCherry control groups. Righting reflex assessments were recorded 1-, 2-, 3-, and 4-weeks after AAV injections for isoflurane or 2 weeks after injection for sevoflurane. Observers performing righting reflex assessments were blinded to the transfected virus. Righting reflex assessments were performed during the active phase, ranging from ZT13 to ZT18.

### Adhesive sticker removal test

PZ^GABA^-ablated or mCherry control mice underwent 3 days of training for sticker removal during the second week after AAV transfection.^51^^,^^52^ A 0.25 inch sticker was applied to the snout, and the latency to remove the sticker was recorded. Successful training was defined as two consecutive trials with removal within 10 s. Three weeks after AAV transfection, mice were exposed to isoflurane 1.2 vol% for 2 h, and the latency between the end of isoflurane exposure and sticker removal was again measured three times in each animal with a minimum of 24 h between testing days.

### Electroencephalogram and electromyogram recordings during isoflurane exposure

PZ^GABA^-ablated or mCherry control mice received EEG and EMG implantations and were recorded at 1000 Hz using a 32-channel headstage (Intan Technologies, Los Angeles, CA, USA) as previously described.^43^^,^^51^^,^^53^ Four weeks after surgery, EEG recordings were performed during isoflurane exposure. Mice were exposed to four 30-min steps: breathing no anaesthetic, followed by isoflurane 0.3 vol%, 0.6 vol%, and 0.9 vol% in a gas-tight recording chamber submerged in a 37ºC water bath. The final 15 min of each exposure were used for EEG spectral analysis. Each 5-s, non-overlapping window containing burst suppression was identified manually by a scorer blinded to group assignment and excluded from spectral estimates. Manually marked epochs with EEG burst suppression were separately scored for the fraction of isoelectric time as a function of anaesthetic concentration.

### EEG/EMG recordings for sleep–wake assessment

To minimise potential confounding effects of repeated anaesthetic exposures, sleep–wake assessments were performed in a distinct cohort of Vgat-IRES-Cre;Ai6 mice. During a single surgery, mice received AAV microinjections targeting the PZ as described above and underwent EEG/EMG implantation.^54^ After a 3-week postsurgical recovery, mice were habituated to the cable tether for 24 h in singly housed recording cages, and 48 h of EEG/EMG recordings were collected. Thereafter, mice underwent 6-h sleep deprivation with gentle handling^55^ (ZT0–ZT6), followed by an additional 18-h EEG/EMG recordings (ZT6–ZT0). Signals were sampled at 256 Hz using Grass Gamma Software (AstraNova Inc., West Warwick, RI, USA) and amplified (20,000×). EEG was bandpass filtered from 0.1 to 100 Hz. EMG was bandpass filtered from 10 to 100 Hz. EEG/EMG recordings were classified in 4-s non-overlapping epochs using Sleep Learning,^56^ a neural network-based open-source programme, as NREM sleep, REM sleep, or wakefulness. Sleep Learning's classification accuracy has been evaluated by multiple groups,^56^ including ours (Supplemental Table S1).

### Assessment of sleep and wakefulness

Wakefulness, NREM sleep, and REM sleep were scored using 4-s epochs and averaged over the 48-h baseline recording. The total duration of time spent in each state was computed across multiple time scales, including across 2-h bins, 24-h periods, and the 12-h lights-on/light-off periods. We implemented a spike-and-slab analysis to quantify microstructural changes in sleep–wake states that conventional analysis can miss.^57^ The spike-and-slab model derives three key measures: (1) total number of bouts, (2) proportion of short bouts (‘spike’, bouts <40 s), and (3) average duration of long bouts (‘slab’, bouts >40 s). We also performed a frequency-based analysis to determine if there are differences in spectral power after PZ^GABA^ ablation compared with mCherry control mice. EEG spectra were computed via fast Fourier transformation using Somnologica Science software (Embla Recording Systems, Broomfield, CO, USA) with a 1 Hz resolution and a 4-s window size. Delta frequency was defined as 1–4 Hz. Sleep homeostatic response was assessed by calculating the delta power averaged over the first 225 four-second NREM sleep epochs scored by Sleep Learning during recovery sleep after 6 h of sleep deprivation as previously described.^58^ Delta power was normalised to baseline delta power, which was the average delta power over the last 4 h of the lights-on period (ZT8–ZT12) on the day before undergoing sleep deprivation. The increase in delta power is a measure of sleep homeostatic response.^59^^,^^60^ To confirm the PZ^GABA^-ablated effects on isoflurane sensitivity, we measured individual righting reflexes in a subset of PZ^GABA^-ablated and mCherry control mice after sleep–wake assessments.

### Efficacy of PZ^GABA^ ablation

After righting reflex, adhesive tape removal testing, EEG assessments of isoflurane sensitivity, or sleep–wake assessments, PZ^GABA^ neuronal number was determined by counting parafacial neurones expressing cytosolic ZsGreen.^32^^,^^61^ PZ^GABA^ neuronal density was calculated as the number of PZ^GABA^ neurones divided by the area of PZ (mm^2^). Mean densities of PZ^GABA^ neurones from the PZ^GABA^-ablated and mCherry control groups were used to calculate the percentage of ablation. Except for data shown in Fig. 2e, PZ^GABA^-ablated mice with more than 50% of survival of PZ^GABA^ neurones (*n*=8/44 mice) were excluded *post hoc* from behavioural or EEG analyses.

### Statistical analysis

Data are summarised as mean (sd) for continuous data and frequencies and percentages for categorical data. Normality was assessed using the Shapiro–Wilk test. Nonparametric tests were used with non-normally distributed data. Unpaired *t*-tests were used to compare the means of PZ cell numbers and the ratio between isoflurane- or oxygen-exposed mice and to analyse PZ^GABA^ density, epochs containing EEG suppression, time spent in each state for 24 h during lights-on/off, relative delta power change during recovery sleep, and the isoflurane righting reflex before EEG measurements during isoflurane exposures between PZ^GABA^-ablated and mCherry control mice. The Mann–Whitney *U*-test was used to compare the means of c-Fos positive neurones in PZ and the spike-and-slab analysis. Isoflurane righting reflex assessments, latency to sticker removal time during training sessions, and normalised delta power during isoflurane exposures were analysed using a repeated-measures two-way analysis of variance (ANOVA) to evaluate the effects of time, virus (PZ^GABA^-ablated *vs* mCherry control mice), and their interaction, with *post hoc* Šídák's multiple-comparison correction. For sevoflurane, a repeated-measures two-way ANOVA was used to evaluate the effects of time, virus (PZ^GABA^-ablated *vs* mCherry control mice), and their interaction, with Fisher's least significant difference correction for multiple comparisons. Pearson's correlation coefficient was computed to assess the relationship between PZ^GABA^ density and sensitivity to isoflurane at 4 weeks. Differences in adhesive sticker removal time across the PZ^GABA^-ablated and mCherry control groups were computed using bootstrap resampling with replacement. For each resampled distribution (10,000 bootstraps for each group), we computed the observed mean difference in adhesive sticker removal times and compared it with a null distribution where sticker removal times across the two groups were assumed to be the same. An empirically calculated *P*-value was derived from the proportion of bootstrap mean differences that were at least as large as the observed difference. EEG biopotentials were processed and analysed in MATLAB using the Signal Processing Toolbox and the Statistics and Machine Learning Toolbox. Raw EEG signals were high-pass filtered (0.5 Hz cutoff frequency) before multitaper spectral estimation^62^ (18 tapers). Then, 95% confidence intervals about the mean of normalised power (per spectral window) were computed across individuals. Analysis of sleep–wake durations in 2-h bins was performed using a repeated-measures two-way ANOVA to evaluate the effects of time (2-h bins) and virus (PZ^GABA^-ablated *vs* mCherry control mice), and their interaction, with *post hoc* Šídák's multiple-comparison test correction. The following notation was used to denote *P*-values: ∗*P*<0.05; ∗∗*P*<0.01; ∗∗∗*P*<0.001; and ∗∗∗∗*P*<0.0001. Analyses were performed using MATLAB 2023b (MathWorks, Natick, MA, USA), Prism v8.0 (GraphPad, La Jolla, CA, USA), and Stata/SE 14.2 (StataCorp LLC, College Station, TX, USA).

## Results

### Isoflurane exposure increases c-Fos expression in PZ^GABA^ neurones

Vgat-IRES-Cre;Ai6 mice were exposed to a hypnotic dose of either isoflurane1.2 vol% in 100% oxygen or 100% oxygen only for 2 h (Fig. 1a and b). There were no significant differences in the number of PZ^GABA^ neurones (*P*=0.50; Fig. 1c). Mice exposed to isoflurane showed a significant increase in nuclear c-Fos expression in the PZ in both green-labelled GABAergic and unlabelled non-GABAergic neurones compared with mice exposed to oxygen control (isoflurane 37.4 [14.4] and oxygen 13.2 [3.2], *P*=0.001; Fig. 1d). Isoflurane exposure also led to a 3.6-fold increase in the percentage of Vgat and c-Fos double-positive neurones in the PZ (isoflurane 8.6% [4.2%] and oxygen 2.4% [1.6%], *P*=0.002; Fig. 1e). Conversely, exposure to the non-immobiliser 1,2-dichlorohexafluorocyclobutane, which is predicted to cause hypnosis based on its chemical structure but does not,^45^ failed to induce a significant increase in c-Fos expression in PZ^GABA^ neurones (Supplemental Fig. S2). These results are consistent with the activation of PZ^GABA^ neurones during isoflurane-induced hypnosis.

**Fig 1:**
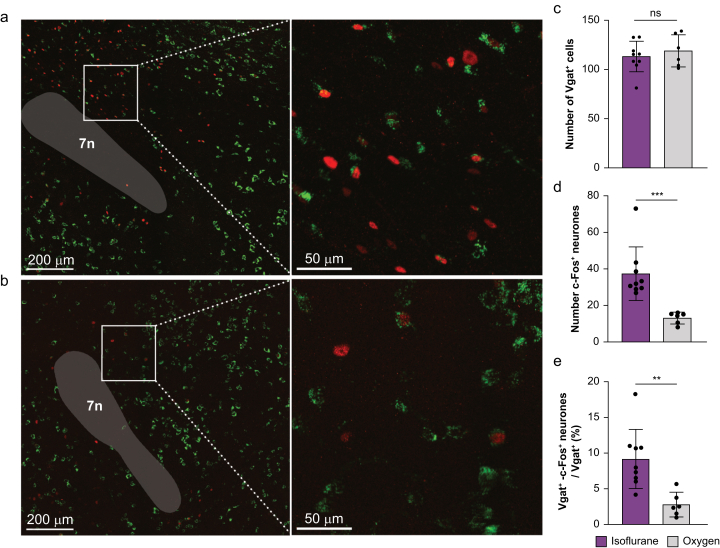
Isoflurane activates PZ^GABA^ neurones. Adult Vgat-IRES-Cre;Ai6 mice were subjected to 2-h exposures to either (a) isoflurane 1.2 vol% (*n*=9) or (b) 100% oxygen (*n*=6). Representative confocal images showing the distribution of Vgat-positive neurones (green) and c-Fos positive neurones (red) in the parafacial zone (PZ). (c) Total number of Vgat-positive neurones was unchanged in the PZ by isoflurane exposure. (d) Number of c-Fos-positive PZ neurones was increased by isoflurane. (e) Percentage of PZ neurones double-positive for Vgat and c-Fos was increased by isoflurane. Data presented as mean (sd). ∗∗*P*<0.01, ∗∗∗*P*<0.001. Data were analysed using an unpaired *t*-test. 7n, facial nerve.

### Viral transformation of parafacial zone neurones

We evaluated viral spread using 30 nl of AAV5-EF1a Nuc-flox(mCherry)-EGFP targeting the PZ of Vgat-IRES-Cre;Ai6 mice to optimise transfecting most of PZ^GABA^ neurones without spillover to brainstem nuclei regulating sleep, such as the parabrachial nucleus and locus coeruleus, whose boundaries are as close as 750 to 1000 μm from the centre of the PZ.^36^^,^^63^ This virus drives expression of an EGFP reporter in Cre^+^ nuclei and an mCherry reporter in Cre^–^ nuclei (Supplemental Fig. S3). Transfected neurones were distributed along an ∼405 μm radius (mCherry-positive areas 430.9 [59.2] μm and EGFP-positive areas 379.7 [45.1] μm). Hence, 30 nl injections were chosen to span the PZ and avoid extending into the parabrachial nucleus or locus coeruleus.

### PZ^GABA^ ablation produces behavioural resistance to anaesthetic-induced hypnosis

To determine the effects of PZ^GABA^ ablation on anaesthetic sensitivity, we exposed mice to their sex-specific population EC_50_ anaesthetic dose and assessed righting reflex under steady-state conditions. PZ^GABA^-ablated mice exhibited a significantly increased propensity of remaining in the awake, responsive state compared with mCherry controls (main effect virus F_(1, 29)_=11.45, *P*=0.0021; Fig. 2a). Consistent with this result, there was an increase in the probability of intact righting reflex across time when comparing the two groups (main effect time F_(3.295, 95.56)_=5.490, *P*=0.0011). There was a significant interaction (time × virus F_(4, 116)_=8.468, *P*<0.0001), with no baseline difference in the probability of intact righting reflex between PZ^GABA^-ablated and mCherry control mice (*P*=0.37) but with increased probability of intact righting reflex in PZ^GABA^-ablated *vs* mCherry control mice at 1, 2, 3, and 4 weeks after AAV injections (*P*=0.002, 0.002, 0.03, and <0.001, respectively). We reasoned that the loss of PZ^GABA^ neurones would not be specific for isoflurane but should affect the propensity to become anaesthetised by other general anaesthetics as well. Indeed, PZ^GABA^-ablated mice were also resistant to sevoflurane 2 weeks after transfection compared with mCherry controls (time × virus F_(1, 29)_=4.512, *P*=0.04; Fig. 2b). To confirm the efficacy of ablation, we assessed the density of PZ^GABA^ neurones 4 weeks after AAV injection (Fig. 2c). Mice transfected with the Cre-dependent caspase virus showed a 73% reduction in PZ^GABA^ neurones compared with controls transfected with the Cre-dependent mCherry (46 [20] and 168 [23] cells mm^−2^ for PZ^GABA^-ablated and mCherry control mice, respectively, *P*<0.0001; Fig. 2d). There was a significant inverse correlation between the probability of becoming anaesthetised as a function of the number of PZ^GABA^ neurones (R^2^ 0.4114, *P*<0.0001; Fig. 2e).

**Fig 2:**
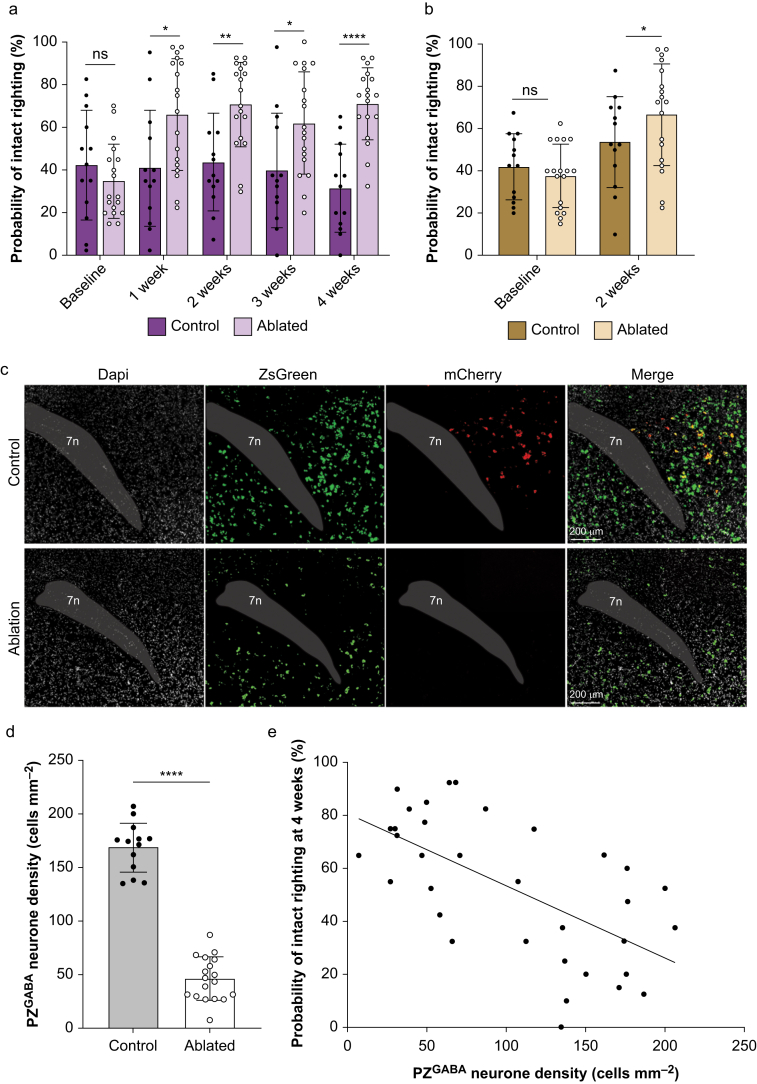
PZ^GABA^-ablated mice become behaviourally resistant to anaesthetic-induced hypnosis. (a) Probability of intact righting reflex was persistently increased across time in isoflurane-exposed PZ^GABA^-ablated mice (*n*=9 males and 9 females) compared with mCherry controls (*n*=6 males and 7 females). (b) PZ^GABA^-ablated mice also showed increased resistance to sevoflurane. (c) Representative parafacial zone (PZ) images of an mCherry control mouse transfected with a Cre-dependent mCherry virus (upper panels) or Cre-dependent caspase-3 virus that ablates GABAergic neurones (lower panels). PZ^GABA^ neurones express a ZsGreen reporter. (d) PZ^GABA^ density was reduced by 73% in PZ^GABA^-ablated mice. (e) There was a significant negative correlation between PZ^GABA^ density and the probability of intact righting reflex during isoflurane exposure at 4 weeks after AAV injection (*n*=35, r=–0.6414, *P*<0.0001). Note that this regression plot also includes PZ^GABA^ ablation mice (*n*=2 male and 2 female) with >100 cells mm^−2^ to better define the relationship between PZ^GABA^ density and isoflurane sensitivity. ∗*P*<0.05, ∗∗*P*<0.01, ∗∗∗∗*P*<0.0001. 7n, facial nerve.

To further confirm behavioural resistance to anaesthetic hypnosis in PZ^GABA^-ablated mice and to bypass confounders of the righting reflex,^64^^,^^65^ a distinct cohort of animals underwent adhesive tape removal testing.^51^ This assay requires mice to exhibit intact sensory input and a coordinated motor response to remove a sticker placed on their snout (Fig. 3a). In the absence of anaesthetic exposure, the PZ^GABA^-ablated and mCherry control groups showed shorter latency to remove stickers as the training sessions progressed without demonstrating any difference in the mean latencies between groups as expected (time F_(2.573, 54.03)_=3.476, *P*=0.03; virus F_(1, 21)_=0.1041, *P*=0.75; and time × virus F_(4, 84)_=0.2155, *P*=0.93; Fig. 3b). This indicates that ablation of PZ^GABA^ neurones did not cause gross facial sensory or forelimb motor deficits. However, consistent with being relatively resistant to a predicted population EC_50_ dose of anaesthetic during steady-state exposures, PZ^GABA^-ablated mice showed reduced latency to remove stickers, indicating faster recovery sensorimotor function after a 2-h isoflurane anaesthetic 1.2 vol% compared with PZ^GABA^ controls (PZ^GABA^-ablated mice: 368 [221–558] s *vs* mCherry control mice 464 [354–644] s, reported as estimated median [25% and 75% IQR]. *P*=0.02; Fig. 3c).

**Fig 3:**
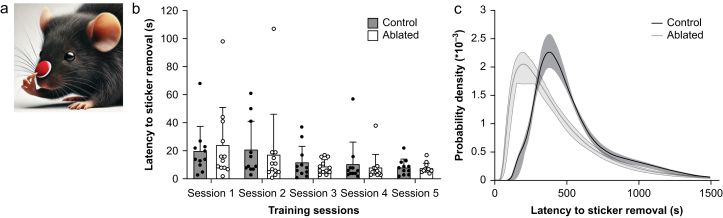
PZ^GABA^-ablated mice emerge from isoflurane and recover sensorimotor function faster than controls. (a) DALL-E3 AI-generated cartoon depicting adhesive tape removal test in which a mouse must have intact trigeminal nerve sensation and forelimb motor cortical function to detect and remove a snout sticker. (b) PZ^GABA^-ablated mice (*n*=12) and control mice (*n*=11) showed no difference in learning and indistinguishably meet the predefined criteria for removing the snout sticker placed during the waking state. (c) After exposure to a 2-h isoflurane 1.2 vol% general anaesthetic, PZ^GABA^-ablated mice exhibited faster emergence and resumed wake-like recovery of sensorimotor function significantly ahead of PZ^GABA^ controls. Solid black and grey curves show the probability density distributions for adhesive tape removal along with the shaded bootstrapped 95% confidence interval estimates.

### Cortical EEG signatures of relative anaesthetic resistance

**Fig 4:**
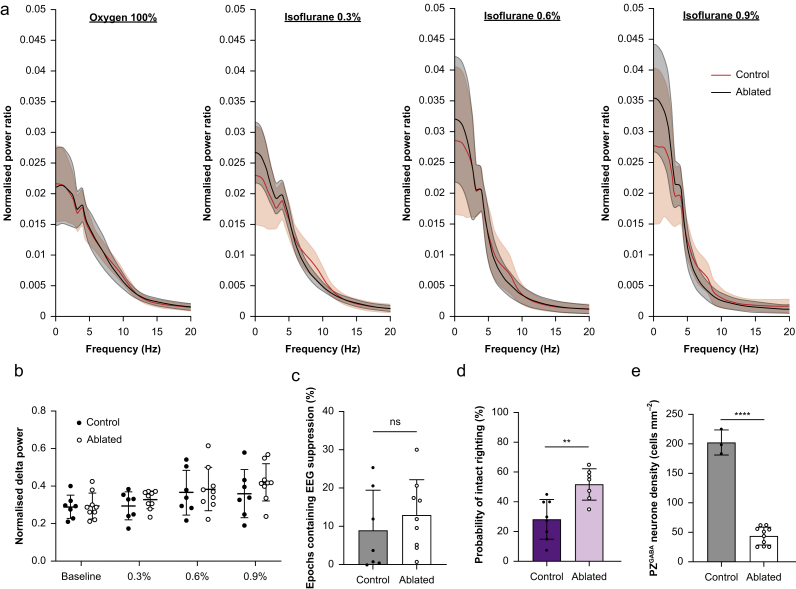
Behavioural anaesthetic resistance is not mirrored in the cortical EEG. (a) Normalised mean (95% CI) spectral power as a function of frequency showed no differences at baseline or during isoflurane 0.3 vol%, 0.6 vol%, and 0.9 vol% between PZ^GABA^-ablated mice (*n*=7 males and 2 females) and PZ^GABA^ mCherry controls (5 males and 2 females) (mean [95% CI] shown). (b) Normalised delta power (1–4 Hz) as a function of group and isoflurane dose does not differ between PZ^GABA^-ablated and mCherry control mice. (c) The percentage of epochs containing EEG suppression is also indistinguishable between PZ^GABA^-ablated and mCherry control mice. (d) Despite the absence of cortical EEG differences, this cohort of PZ^GABA^-ablated mice also exhibited resistance to isoflurane-induced loss of righting compared with PZ^GABA^ mCherry control mice. (e) Accordingly, PZ^GABA^ density was reduced by 78% in this cohort of PZ^GABA^-ablated mice compared with control mice. ∗∗*P*<0.01, ∗∗∗∗*P*<0.0001. Data in (b)–(e) are presented as mean (sd).

We hypothesised that PZ^GABA^-ablated mice exhibit relative resistance to isoflurane based on stereotypical anaesthetic-induced changes in the EEG. There were no spectral differences between ablated and mCherry control mice at baseline (Fig. 4a). Normalised delta power increased as isoflurane concentration increased, but there were no differences in delta power between PZ^GABA^-ablated and control mice (time F_(2.471,34.60)_=8.356, *P*=0.0005; virus F_(1,14)_=0.6074, *P*=0.45; and time × virus F_(3,42)_=0.5282, *P*=0.67; Fig. 4b). Moreover, at high isoflurane doses, there was no difference in the epochs containing EEG suppression between PZ^GABA^-ablated mice (13% [9%]) and mCherry control mice (9% [10%]) (*P*=0.43; Fig. 4c). The failure to find processed EEG evidence of isoflurane resistance in PZ^GABA^-ablated mice was not attributed to viral mistargeting in this third cohort. PZ^GABA^-ablated mice showed comparable behavioural resistance to isoflurane based on righting reflex testing (PZ^GABA^-ablated mice 51.8% [10.6%] *vs* mCherry control mice 28.1% [13.3%], *P*=0.002; Fig. 4d) and comparable ablation efficiency with past cohorts (PZ^GABA^-ablated mice 44 [15] cells mm^−2^ *vs* mCherry control mice 202 [21] cells mm^−2^, *P*<0.0001; Fig. 4e).

### PZ^GABA^ ablation does not alter sleep–wake architecture or homeostatic response to sleep deprivation

**Fig 5:**
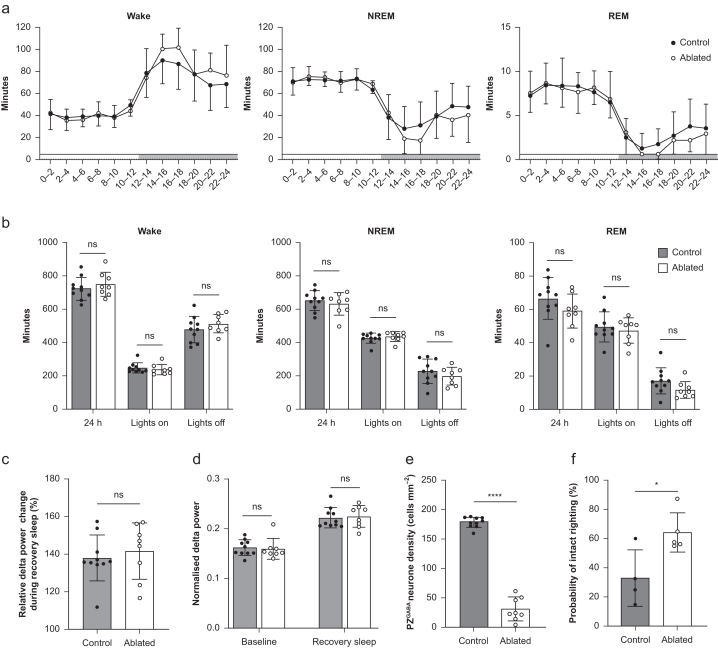
Ablation of PZ^GABA^ neurones does not affect sleep–wake states or the homeostatic drive to sleep. There were no differences in total time spent in states of wake, NREM sleep, or REM sleep between PZ^GABA^-ablated mice (*n*=5 males and 3 females) and mCherry controls (*n*=4 males and 6 females) in (a) 2-h time bins or (b) time spent in each state for 24 h, during the 12-h light cycle, or during the 12-h dark cycle. (c) Relative delta power change during recovery sleep from the baseline was not different between PZ^GABA^-ablated and mCherry control mice. (d) Relative delta power at baseline and during recovery sleep that follows a 6-h sleep deprivation for PZ^GABA^-ablated *vs* mCherry controls. (e) Despite the absence of changes in the architecture of sleep and wakefulness, PZ^GABA^ neuronal density was reduced in this cohort of PZ^GABA^-ablated mice by 83% compared with mCherry controls. (f) As before, with the loss of PZ^GABA^ neurones, this cohort of PZ^GABA^-ablated mice exhibit significant resistance to isoflurane upon righting reflex testing compared with mCherry controls. Data presented as mean (sd). ∗*P*<0.05, ∗∗∗∗*P*<0.0001. NREM, non-rapid eye movement sleep, REM, rapid eye movement sleep.

In a final cohort, we analysed sleep–wake architecture in response to PZ^GABA^ *ablation*. EEG/EMG recordings demonstrated no significant differences in the total amount of NREM sleep, REM sleep, or wakefulness in PZ^GABA^-ablated mice compared with mCherry control mice (Fig. 5a and b) or in the characteristics of sleep/wake bouts quantified through the spike-and-slab method^66^ (Table 1). As sleep pressure alters anaesthetic sensitivity,^13^^,^^67^^,^^68^ we examined whether PZ^GABA^ ablation alters the homeostatic drive for sleep. We sleep-deprived mice for 6 h by gentle handling but found no difference in percentage increase in relative delta power, calculated as delta power over total spectral power, during recovery sleep (*P*=0.59; Fig. 5c). Sleep deprivation was nevertheless effective, as indicated by significant increases in relative delta power during recovery sleep from baseline in both groups (*P*<0.001 for both). However, there were no differences in relative delta power at baseline (*P*=0.81) or during recovery sleep (*P*=0.82) between PZ^GABA^-ablated and mCherry control mice (Fig. 5d). This indicates indistinguishable homeostatic sleep responses between groups after sleep deprivation. In this cohort, AAV-mediated ablation reduced PZ^GABA^ neurones to 17% of mCherry controls 4 weeks after AAV injection (*P*<0.0001; Fig. 5e).

**Table 1:** Spike-and-slab analysis of state transitions. Each transition (wake to NREM, NREM to REM, NREM to wake, and REM to wake) during the lights-on and lights-off periods. Long bouts are defined as episodes lasting ≥40 s. Data are presented as mean (sd). NREM, non-rapid eye movement sleep, REM, rapid eye movement sleep.

		Number of episodes			Fraction of short bouts			Length of long bouts (min)		
		Control	Ablated	*P*-value	Control	Ablated	*P*-value	Control	Ablated	*P*-value
Light-on	Wake to NREM	278.5 (85.6)	252.0 (62.8)	0.31	0.13 (0.04)	0.11 (0.04)	0.32	1.88 (0.63)	2.05 (0.59)	0.44
	NREM to REM	42.4 (11.0)	41.6 (6.2)	0.8	0.24 (0.08)	0.25 (0.09)	0.84	1.44 (0.21)	1.38 (0.18)	0.44
	NREM to wake	236.2 (82.5)	209.9 (59.1)	0.29	0.57 (0.05)	0.59 (0.06)	0.3	1.88 (0.57)	1.93 (0.80)	0.8
	REM to wake	42.3 (11.1)	41.8 (6.1)	0.88	0.68 (0.16)	0.76 (0.14)	0.15	2.27 (1.89)	3.64 (2.95)	0.1
Light-off	Wake to NREM	171.2 (122.1)	152.2 (75.7)	0.59	0.17 (0.07)	0.20 (0.12)	0.34	2.05 (0.90)	2.03 (0.94)	0.93
	NREM to REM	14.5 (5.8)	11.3 (4.4)	0.09	0.30 (0.12)	0.36 (0.16)	0.18	1.49 (0.18)	1.38 (0.32)	0.25
	NREM to wake	157.0 (26.3)	141.5 (73.6)	0.65	0.52 (0.09)	0.46 (0.13)	0.12	12.00 (13.45)	8.61 (5.10)	0.31
	REM to wake	14.4 (5.8)	11.4 (4.4)	0.1	0.76 (0.19)	0.81 (0.08)	0.44	6.91 (7.95)	10.19 (12.30)	0.41

To exclude the possibility that the cohort of mice that underwent sleep–wake assessments might have a functionally distinct ablation, we independently assessed the righting reflex in this sleep study cohort by exposing males from both groups to isoflurane 4 weeks after viral injections. Confirming functionally indistinguishable viral targeting between the cohort of mice for anaesthesia sensitivity and sleep–wake assessments, PZ^GABA^-ablated animals were again found to be more resistant to the hypnotic effects of isoflurane at 4 weeks after AAV microinjections (*P*=0.02; Fig. 5f).

## Discussion

The intersection between anaesthesia and sleep has been an area of intense research, given that both states share a common feature: reversible loss of consciousness. However, the neuronal mechanisms driving these processes and the extent to which anaesthetic hypnosis relies on the recruitment of sleep-promoting neurones remain unclear. This study focused on a group of GABAergic neurones in the PZ, which are implicated in the control of NREM sleep.^31^^,^^32^ We show that PZ^GABA^ neurones express c-Fos when exposed to hypnotic doses of isoflurane, but not to a non-immobiliser, supporting the idea of anaesthetic-induced depolarisation. Moreover, PZ^GABA^ ablation results in robust and durable resistance to anaesthetic hypnosis, with behavioural resistance to isoflurane inversely correlating with the number of surviving PZ^GABA^ neurones.

Sleep-active neurones in the PZ send inhibitory projections throughout the brain, including the parabrachial nucleus.^33^ *In vivo* calcium imaging reveals that parabrachial neurones, which regulate sleep and arousal,^36^, ^37^, ^38^ are less active under anaesthesia but become highly active during recovery.^34^ Chemogenetic activation of parabrachial neurones enhances recovery from isoflurane-induced anaesthesia.^34^^,^^40^^,^^41^ In addition, parabrachial astrocytes also influence sleep and isoflurane anaesthesia.^69^ The relationship between the PZ and parabrachial nucleus predicts a dual role of PZ^GABA^ neurones in regulating both sleep and anaesthesia. This dual role in sleep and anaesthesia has been reported in other regions receiving projections from the PZ, such as the lateral hypothalamus,^70^ substantia innominata,^71^ and pontine reticular nucleus, and the oral part.^72^ However, despite bilateral PZ^GABA^ ablation sufficient to bias the brain behaviourally toward wakefulness against two different inhaled anaesthetics, we did not observe changes in endogenous sleep or wakefulness.

The behavioural phenotype of anaesthesia resistance was not reflected in the cortical EEG. Although exposure to increasing doses of isoflurane increased the fraction of total power in the delta range and induced burst suppression, these stereotypic effects were indistinguishable between PZ^GABA^-ablated and control mice. The parafacial nucleus is immediately adjacent to the vestibular nuclei, whose function is required for intact righting. Arguing against a behavioural confound of righting reflex testing in PZ^GABA^-ablated mice are the following. Firstly, PZ^GABA^-ablated mice reliably exhibit enhanced righting and resist the hypnotic effects of anaesthetics compared with controls, which would be unexpected for an ablation that disrupts vestibular function. Secondly, PZ^GABA^-ablated mice also exhibit faster emergence, as measured by adhesive sticker removal testing. This behavioural assay requires mice exiting anaesthetic states to both sense the sticker on their snout and execute an appropriate forelimb motor response to remove the sticker. In both behavioural tests, the former dependent on hindbrain function and the latter dependent on both the trigeminal nerve and motor cortex, PZ^GABA^-ablated mice exhibit evidence of anaesthetic resistance. Hence, our PZ^GABA^-ablated mice appear to provide another example of unmasking hidden intermediate states that must exist along the pathway from anaesthetic-induced hypnosis to wakefulness in which the behavioural and cortical EEG signatures of hypnosis can be incongruent.^64^^,^^73^^,^^74^

The lack of change in sleep–wake state regulation and homeostatic response to sleep deprivation after PZ^GABA^ ablation was unexpected. Despite previous evidence of the PZ's role in NREM sleep regulation,^31^^,^^32^ an *in vivo* electrophysiological study documented PZ neurones with sleep-active or NREM-selective discharge rates.^61^ However, in a subsequent *in vivo* study, none of the 125 PZ neurones recorded fired preferentially during NREM sleep.^75^ Hence, the actual contribution of PZ^GABA^ neurones to NREM sleep might be less certain than initially thought. Arguing for the potential integrated function of both GABAergic and non-GABAergic parafacial neurones is the finding that less selective PZ ablations created by an orexin–saporin toxin that destroys all cells expressing orexin receptors increase daily wakefulness. Further, the observation that c-Fos expression in the PZ during NREM sleep is not found only in GABAergic cells^31^ is consistent with our result of c-Fos expression during isoflurane exposure (Fig. 1e). These findings suggest that non-GABAergic cells in the PZ play an important role in both sleep and anaesthesia. Another possible explanation for why our PZ^GABA^-ablated mice were not more awake relates to ablation efficiency. We used a smaller volume of AAV to ablate 70–80% of PZ^GABA^ neurones. Previous work^31^ did not quantify ablation efficacy, but the use of larger viral volumes could have resulted in more complete ablation of PZ^GABA^ neurones, with potential extension outside of the PZ. It is possible that a larger ablation approaching 100% or a less specific ablation of both GABAergic and non-GABAergic neurones might have produced effects on NREM sleep, as hypothesised.

Mechanistically, NREM sleep and anaesthesia share overlapping neural circuitry, which might partially explain the similarities observed in the cortical EEG of individuals during NREM sleep and anaesthetic-induced hypnosis. This includes the alpha spindles phase coupled with low delta power and the appearance of K-complexes.^76^ However, despite these similarities, NREM sleep and general anaesthesia remain distinct states. Sleep plays a critical role in memory consolidation and retrieval,^11^ whereas anaesthesia can impair memory formation.^77^ NREM sleep enhances immune function, such as enhanced response to vaccinations and reduced risk of infection,^12^ whereas anaesthesia can suppress immune function.^78^ These physiological divergences suggest underlying differences in circuit dynamics between the two states, indicating that although general anaesthetics engage aspects of sleep–wake circuitry, they do not perfectly replicate the neurophysiology of NREM sleep.

At the circuit level, dissociation between sleep and anaesthesia must therefore exist. Recent work illustrates one such dissociation,^9^ showing that chemogenetic modulation of GABAergic neurones in the median preoptic hypothalamus or glutamatergic neurones in the ventrolateral preoptic hypothalamus is sufficient to affect sleep–wake states but fails to alter the latency to enter or exit isoflurane anaesthesia. Conversely, our study demonstrates the opposite dissociation, in which partial ablation of PZ^GABA^ neurones induces relative resistance to general anaesthesia without an expected increase in wakefulness. This finding challenges the assumption that subcortical circuits engaged during general anaesthesia necessarily modulate the endogenous control of sleep–wake states in a congruent manner.

Our study has limitations. We ablated PZ^GABA^ neurones instead of using chemogenetic or optogenetic approaches that provide reversible temporal control over the neuronal activity. Nevertheless, the irreversible ablation highlights an important uncoupling of drug-induced endogenous modulation of arousal. We studied two volatile anaesthetics and attempted to contrast their actions with a non-immobiliser. However, we did not comprehensively apply all three drugs to all endpoints and depended on isoflurane as a standard. Whether other general anaesthetics activate PZ^GABA^ neurones and whether PZ^GABA^ ablations would produce resistance to other drugs remain unknown. We applied two different behavioural measures to evaluate anaesthetic hypnosis but recognise a growing list of alternative ways to phenotype the anaesthetic state.^64^ Finally, we did not assess whether the effect of PZ^GABA^ ablation on anaesthetic sensitivity arises directly or indirectly as a result of altered PZ^GABA^ afferent or efferent innervation, such as from the parabrachial nucleus. Given the complexity of the neuronal network, it is important to study the cellular diversity within the PZ at the single-cell level, rather than relying solely on ablation. This is a direction for future research.

Understanding the convergent and divergent pathways of sleep and anaesthesia is crucial, as it could illuminate the neurobiology of both states. We surprisingly uncovered more robust roles for PZ^GABA^ neurones during anaesthesia than upon sleep regulation. Unravelling the complexities of neural networks governing consciousness during physiologic (sleep), pharmacologic (anaesthesia), and pathologic (coma) perturbations remains an area for further scientific inquiry.

## Authors’ contributions

Conceptualisation, methodology, supervision, resources, and funding acquisition: TI, AIP, MBK

Data acquisition, analysis, and interpretation: TI, AZW, BTK, SLR, JL, MI, XG, NS, AIP, MBK

Writing original draft: TI

Manuscript review and editing: TI, AZW, BTK, SLR, JL, MI, XG, NS, AIP, MBK

## Funding

US 10.13039/100000002National Institutes of Health (R35 GM1511166 to TI, T32 HL007713 to TI, T32 GM112596 to AZW, T32 HL007953 to SLR, P01 HL160471 to AIP, R01 GM088156 to MBK, R01 GM151556 to MBK); American Thoracic Society ASPIRE Fellowship (to TI).

## Declaration of interest

The authors declare no competing interests.

## Figures and Tables

**figs1:**
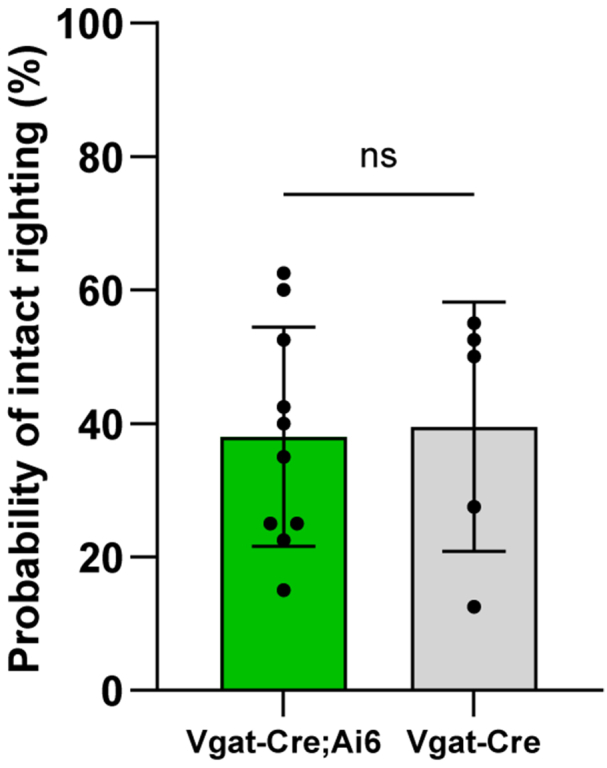


**figs2:**
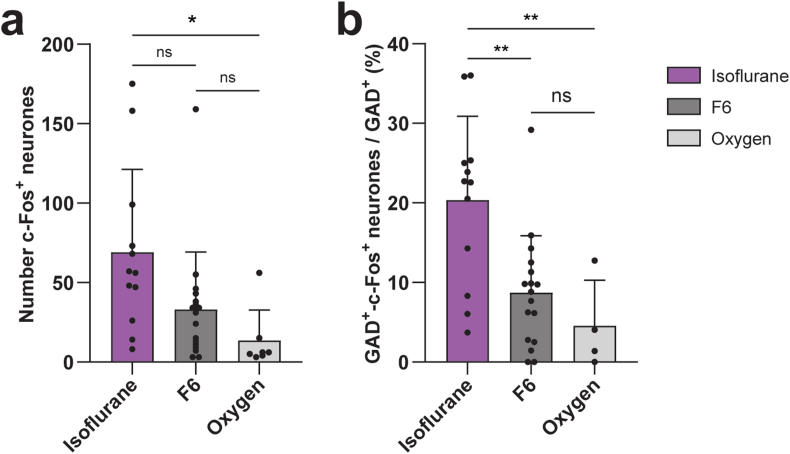


**figs3:**
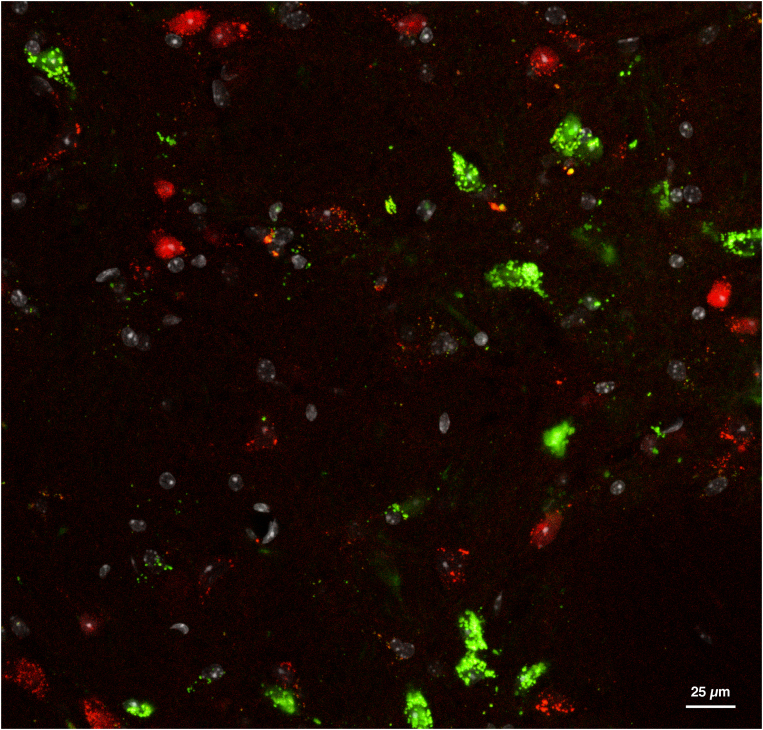


**figs4:**