# Supplementary material for: Parafacial GABAergic neurone ablation induces behavioural resistance to volatile anaesthetic-induced hypnosis without reducing sleep
Source: Br J Anaesth. 2025 Apr 15;134(6):1696–708. doi: 10.1016/j.bja.2025.02.035 (PMC12106870; doi:10.1016/j.bja.2025.02.035)
Supplement: Multimedia component 1 [file mmc1.docx]

**Supplemental Figure 1. No difference in sensitivity to isoflurane measured by the probability of righting reflex at baseline between the two genotypes.** As expected, there was no difference in isoflurane sensitivity as measured by the population probability of intact righting reflex between Vgat-IRES-Cre;Ai6 that express a fluorescent ZsGreen reporter in GABAergic neurons and Vgat-IRES-Cre that did not inherit the ZsGreen gene.

**Supplemental Figure 2. Unlike exposure to a hypnotic dose of isoflurane, exposure to the non-immobilizer, F6, does not increase c-Fos in PZ^GABA^ neurones.** Adult male C57BL/6J mice were subjected to 2-hour exposures to either 1.2% isoflurane, 3.2% non-immobilizer 1,2-dichlorohexafluorocyclobutane (F6) mixed in 100% oxygen, or 100% oxygen. (a) The number of c-Fos positive neurones in the PZ was increased by isoflurane exposure compared to oxygen control. There was no change between F6 and oxygen control. (b) The percentage of GAD and c-Fos double-positive neurones within total GAD-positive neurones in the PZ was significantly higher in isoflurane group than F6 and oxygen controls. Once again, there was no difference between oxygen and F6 controls. Data presented as mean(SD). Data were analysed using one-way ANOVA with a *post hoc* Turkey’s multiple comparison test. *P<0.05, **P<0.01.

**Supplemental Figure 3. Fluorescent microscopic image of AAV5-EF1a Nuc-flox(mCherry)-EGFP transfection.** Vgat-IRES-Cre;Ai6 mice (n=4) that received bilateral PZ microinjections of AAV5-EF1a Nuc-flox(mCherry)-EGFP “colour-switch virus”, exhibit nuclear mCherry expression in Cre^-^ neurones (red) and nuclear expression of EGFP Cre^+^ neurones (green). Note: The Cre^+^ neurones also express cytosolic ZsGreen in Vgat-IRES-Cre;Ai6 mice. Shown in shades of grey are DAPI-stained nuclei. Note that not every neuron has been transfected.

**Supplemental Table 1. Confusion matrix and statistics to assess accuracy of auto EEG scoring by Sleep Learning.** To evaluate the accuracy of Sleep Learning autoscoring, EEG data recorded on 28 male and female C57BL6J mice were used to compare Sleep Learning autoscoring against manual scoring (TechIndia). The overall epoch-to-epoch agreement between the machine learning and human scorers was 92.6%, with 97.3% agreement in wake scoring, 89.7% agreement in NREM, and 84.9% agreement in REM. NREM, non-rapid-eye-movement sleep, REM, rapid-eye-movement sleep.
